# Simultaneous dimension and tolerance design for robot manipulator considering cost and positioning accuracy reliability

**DOI:** 10.1007/s00170-026-17862-8

**Published:** 2026-03-18

**Authors:** Zhiwei Zhao, Yan Jin, Paul Goodall, Andrew West, Mark Price

**Affiliations:** 1 School of mechanical and aerospace engineering, Queen’s University Belfast, Belfast, UK; 2 Wolfson school of mechanical, Electrical and Manufacturing Engineering, Loughborough university, Loughborough, UK

**Keywords:** Robot design, Position accuracy, Tolerance allocation, Manufacturing cost

## Abstract

Tolerance allocation is an important design step for determining robot accuracy and directly affecting manufacturing cost. However, existing methods typically consider dimension synthesis first before tolerances are allocated, which neglects the manufacturability constraints arising from the dependency between part size and achievable tolerance grades. This often leads to costly iterations between design and manufacturing and increasing manufacturing cost. To address this issue, an integrated tolerance allocation and dimensional synthesis method of robot design is proposed for optimizing both positioning accuracy reliability and manufacturing cost. The method simultaneously optimizes joint dimensions and corresponding tolerances by formulating a cost function that captures the relationship between dimensional parameters, robot end-effector accuracy reliability, tolerance-grade rules, and manufacturing cost. Additionally, a matrix-based Monte Carlo simulation (MCS) method is introduced to accelerate evaluation workspace-wide reliability under tolerance uncertainty. NSGA-II multi-objective optimization algorithm is employed to find the Pareto front of the optimal solutions. A case study of a surgical robot is taken to demonstrate the effectiveness of the proposed approach. Results show that the proposed method can reduce 22% of manufacturing cost while achieving better positioning accuracy reliability compared to the traditional tolerance allocation method, and the speed of matrix-based MCS method is improved by 400 times compared to the point-based MCS method.

## Introduction

Robotic manipulators are widely used in industrial and medical applications, where their structural design must satisfy multiple performance objectives, such as workspace coverage, load capacity, positioning accuracy, stiffness, and manufacturing cost. These objectives are often interdependent and conflicting, requiring careful trade-offs during the design process. Among them, positioning accuracy is a key performance metric, especially in applications demanding high precision, where the precise location of the end-effector tip is the critical factor determining task success [1, 2]. Unacceptable robotic position error may result in severe consequences, including but not limited to manufacturing quality deterioration and potential patient safety hazards in medical applications [3]. Therefore, positioning accurate design is fundamental to development and manufacturing of robots, directly influencing their overall performance and reliability. The positioning accuracy of robots is influenced by factors spanning the entire design, manufacturing, and assembly processes, as well as the performance of the motion control system. Among these factors, errors resulting from dimensional deviations and assembly tolerances are major contributors to positioning inaccuracy [4].

In traditional design of robot manipulators, dimensional synthesis mainly aims for maximum workspace volume, while accuracy synthesis is taken as secondary objective after dimensions are fixed. This essentially place the workspace volume objective as the priority rather than the accuracy. Therefore, this method may not be suitable to the robot design for accuracy applications, in which accuracy is much more crucial than the workspace volume, or the workspace volume is prescribed for the tasks (such as eye surgery) rather than maximized. In addition, both link dimensions and manufacturing/assembly tolerances will combinatorially affect manufacturing cost. The traditional 2-step approach will miss the opportunity to optimize the combined effects of both dimensions and tolerances on manufacturing cost. This highlights the need for an integrated design method that simultaneously considers dimensions and tolerances to achieve reliable accuracy with cost-effective manufacturability.

To evaluate how dimensional and assembly errors affect robotic positioning accuracy, two main types of modeling approaches have been developed: analytical methods and numerical simulation methods. Analytical methods typically employ differential kinematics and sensitivity analysis to derive explicit relationships between link errors and the positional accuracy of end effector. Amanpreet et al. [5, 6] analyzed the sensitivity of Remote Center of Motion (RCM) mechanisms and proposed appropriate structural constraints to ensure accuracy. Similarly, Smits et al. [7] and Shi et al. [8] conducted detailed kinematic modeling and reliability analysis for RCM systems, focusing on end-effector position errors. On the other hand, numerical simulation methods use Monte Carlo simulation (MCS) to statistically evaluate the effect of multiple uncertain parameters across a range of configurations. However, its computational cost is often prohibitive due to the inherently large number of random samples required. Therefore, approximation methods, such as low-order statistical moments, have been proposed to accelerate the evaluation process while maintaining acceptable accuracy levels. Wu et al. [9] developed a reliability analysis framework based on the statistical moment similarity of positional errors, allowing efficient evaluation of positioning accuracy by calculating lower-order statistical moments. Cao et al. [10] introduced an evidence theory-based method to model epistemic uncertainty and parameter correlation, providing a robust positioning accuracy reliability assessment for industrial robots. Xu et al. [11] proposed an uncertain hybrid tolerance allocation (UHTA) approach for a parallel robotic mechanism. Furthermore, several studies employed saddle point approximation and differential kinematics to model the propagation of joint dimensional errors to the end-effector, achieving accurate reliability estimation with reduced computational cost [12–14]. While these approximation methods reduce the computational burden on individual configurations, they still require evaluations across a large number of configurations throughout the entire workspace to obtain reliable system-level accuracy estimates.

To effectively control position accuracy, tolerance allocation serves as a key strategy in robot design and manufacturing. Numerous studies have been conducted to optimize tolerance allocation to ensure robot position accuracy. These methods consider not only positioning accuracy but also factors such as manufacturing cost, machining time, and production feasibility. Thang et al. [15] investigated the relationship between the cost and tolerance of machined robot parts, highlighting how tighter tolerances lead to exponentially increasing manufacturing costs. Trang et al. [16] transformed the tolerance allocation problem into a nonlinear multi-variable optimization model and solved it using the generalized reduced gradient algorithm, focusing on meeting accuracy targets. Similarly, Peng et al. [17] extended the concurrent tolerance allocation framework by incorporating geometrical tolerances into multi-process machining parts, enhancing the control over assembly precision. Gao et al. [18] proposed a tolerance allocation method for 6- Degrees of Freedom (DOF) manipulators based on Denavit–Hartenberg (D-H) parameters, utilizing an extremum error model to balance positioning accuracy and manufacturability. Lenin et al. [19] proposed a two-step optimization method to minimize both tolerance cost and machining time in complex assemblies. Maroua et al. [20] developed a genetic algorithm-based tolerance allocation method that integrates manufacturing difficulty prediction, aiming to reduce production costs while ensuring functional and quality requirements. Wang et al. [21] further expanded the scope by incorporating production rate planning and waste minimization into the tolerance allocation process. Similarly, Wang et al. [22] proposed an accuracy synthesis approach for parallel mechanisms that considers allowable accuracy thresholds within the workspace to balance positioning performance and manufacturing cost. Huang et al. [23] proposed an optimal tolerance design approach for robot manipulators by integrating differential kinematics, eigen-decomposition, and genetic algorithms to minimize failure probability while considering manufacturing cost constraints. Recent work has also shown that systematic tolerance optimization can significantly reduce manufacturing cost while maintaining functional performance [24]. While tolerance allocation methods have been extensively studied, they are often performed based on pre-defined dimensional parameters, without considering how the selection of these parameters influences achievable tolerance grades and overall production cost.

In parallel, a number of studies have focused on the dimensional optimization of robotic manipulators, primarily aiming to improve workspace coverage, motion performance, and mechanical properties. For instance, Smits et al. [7] proposed a 2-DoF RCM mechanism and optimized its structure by considering workspace coverage, potential energy, manipulability, and reflected stiffness. Qiu et al. [25] developed a parallel surgical robot with a remote center of motion and employed a multi-objective optimization approach using quantum particle swarm optimization to enhance the effective workspace volume and global dexterity. Similarly, Wang et al. [26] utilized a multi-objective optimization method to adjust the dimensional parameters of a robotic manipulator, aiming to enlarge the motion range within a specific target area while reducing potential collisions between mechanical arms.

Despite significant progress in tolerance allocation and dimensional optimization, most existing methods treat dimensional design, tolerance allocation, and manufacturing feasibility as separate processes [27]. In the design phase, tolerances are typically assigned based on predefined link dimensions without considering the manufacturability constraints imposed by their interdependence. This can result in assigning overly tight tolerances to large-sized links, which significantly increases machining difficulty due to the inherent relationship between dimension and achievable tolerance. As a consequence, repeated iterations between the design and manufacturing stages are often required to meet task and manufacturing requirements, leading to higher production costs and reduced efficiency.

**Fig. 1:**
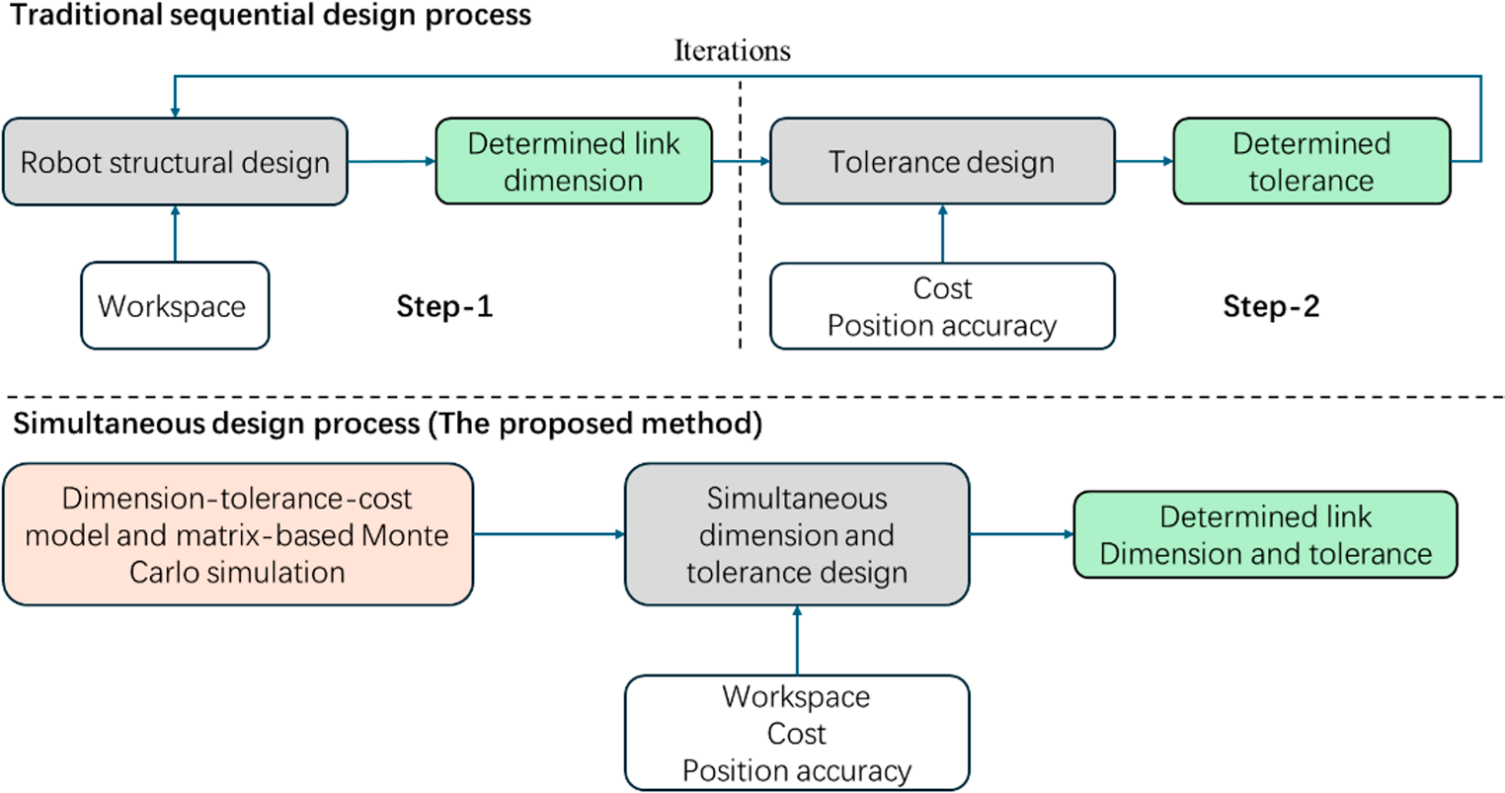
The flowchart of simultaneous dimension and tolerance design process

To address these limitations, this paper proposes a simultaneous dimension and tolerance design method of robotic manipulators, as shown in Fig. 1. The proposed approach jointly optimizes joint dimensions and corresponding tolerances by establishing a cost function that reflects the relationship between dimensional parameters, tolerance-grades and manufacturing cost. An error propagation model is developed to quantify the effect of joint dimensional and assembly errors on end-effector positioning accuracy. To enable efficient evaluation across the entire workspace, a matrix-based Monte Carlo simulation method is introduced, achieving significant computational speedup compared to conventional sampling approaches. Based on these models, an optimization method is formulated to minimize total manufacturing cost while satisfying specified positional accuracy reliability requirements. The effectiveness of the proposed method is demonstrated through a case study on a surgical robot, which achieves a better balance between positioning accuracy, cost and manufacturability than traditional tolerance allocation approaches.

## Positioning accuracy reliability model

This section establishes the modeling process for evaluating the positioning accuracy reliability of robots. It begins by describing the kinematics using the D-H parameter method, followed by a Jacobian-based error propagation model that relates joint parameter deviations to end-effector positional errors. A probabilistic approach is then introduced to quantify the positioning accuracy reliability, defined as the probability that the end-effector remains within a specified accuracy threshold across the workspace.

### Forward kinematic modeling using the D-H parameter method

In the ideal scenario, the kinematic behavior of a robot manipulator is described using a deterministic model based on known geometric parameters. In this study, the D-H method parameter is adopted to model the forward kinematics of the robot [23]. By assigning coordinate frames to each joint, the relative transformation between adjacent links is defined through four parameters: joint angle ${\theta}_{i}$, link length ${l}_{i}$, link offset ${d}_{i}$, and link twist ${\alpha}_{i}$. Specifically, the homogeneous transformation matrix from the $(i-1)-th$ to the $i-th$ coordinate frame is expressed as:

1 $$\boldsymbol{T}_{i-1}^{i}=\left[\begin{array}{cccc}\cos{\theta}_{i}&-\cos{\alpha}_{i}\sin{\theta}_{i}&\sin{\alpha}_{i}\sin{\theta}_{i}&{l}_{i}\cos{\theta}_{i}\\\sin{\theta}_{i}&\cos{\alpha}_{i}\cos{\theta}_{i}&-\sin{\alpha}_{i}\cos{\theta}_{i}&{l}_{i}\sin{\theta}_{i}\\0&\sin{\alpha}_{i}&\cos{\alpha}_{i}&{d}_{i}\\0&0&0&1\end{array}\right],i=\mathrm{1,2},\ldots,K,$$

where $K$ is the number of joints. The complete transformation from the base frame to the end-effector frame for a serial robot is obtained by successive multiplication of each transformation of joint:

2 $${\boldsymbol{T}}_{0}^{I}=\prod_{i=1}^{K}{\boldsymbol{T}}_{i-1}^{i}=\left[\begin{array}{cccc}{n}_{ix}&{s}_{ix}&{l}_{ix}&{p}_{ix}\\{n}_{iy}&{s}_{iy}&{l}_{iy}&{p}_{iy}\\{n}_{iz}&{s}_{iz}&{l}_{iz}&{p}_{iz}\\0&0&0&1\end{array}\right],$$

where ${n}_{i}$, ${s}_{i}$ and ${l}_{i}$ are the orientation vectors, and ${\boldsymbol{p}}_{i}={\left({p}_{ix},{p}_{iy},{p}_{iz}\right)}^{\mathrm{T}}$ represents the end-effector position in the base frame.

### Kinematic error propagation modeling

However, in practical robotic manipulator applications, manufacturing tolerance deviations introduce unavoidable uncertainties to kinematic parameters. These uncertainties propagate through the kinematic chain, leading to deviations between the actual and target positions of the end-effector, which is the positional error.

Let ${\boldsymbol{P}}_{r}=({X}_{r},{Y}_{r},{Z}_{r}{)}^{\mathrm{T}}$ and ${\boldsymbol{P}}_{t}=({X}_{t},{Y}_{t},{Z}_{t}{)}^{\mathrm{T}}$ denote the actual and target positions of the end-effector. The positional error $\varDelta\boldsymbol{P}$ is defined as,

3 $$\varDelta\boldsymbol{P}={\boldsymbol{P}}_{r}-{\boldsymbol{P}}_{t}=({\Delta}\mathrm{X},{\Delta}\mathrm{Y},{\Delta}\mathrm{Z}{)}^{\mathrm{T}}.$$

Based on the kinematic model, the positional error ${\Delta}\boldsymbol{P}$ can be expressed as a function of the kinematic parameter errors, including joint angle errors $\varDelta{\theta}_{i}$, link length errors $\varDelta{l}_{i}$, joint offset errors $\varDelta{d}_{i}$, and link twist errors $\varDelta{\alpha}_{i}$ from each joint$i$. Let ${\boldsymbol{q}}_{i}=(\varDelta{\theta}_{i},\varDelta{l}_{i},\varDelta{d}_{i},\varDelta{\alpha}_{i}{)}^{\mathrm{T}}$ represents the kinematic parameter errors associated with the $i-th$ link in the kinematic chain, the error propagation model is formulated as,

4 $${\Delta}\boldsymbol{P}=\varSigma\left({\boldsymbol{J}}_{i}\cdot{\boldsymbol{e}}_{i}\right)=\varSigma\left({\boldsymbol{J}}_{i}\cdot{\boldsymbol{I}}_{i}\cdot{\boldsymbol{q}}_{i}\right)=\varSigma\left({\boldsymbol{h}}_{i}\cdot{\boldsymbol{q}}_{i}\right),$$

where ${\boldsymbol{J}}_{i}$ is the equivalent transformation matrix, and ${\boldsymbol{e}}_{i}$ is the pose errors. ***I****i* is the error transformation matrix relating joint parameter errors to pose errors. ${\boldsymbol{h}}_{i}$ is the positional error coefficient matrix.

The equivalent transformation matrix ${\boldsymbol{J}}_{i}$ is expressed as,

5 $${J}_{i}=\left[\begin{array}{cccccc}{n}_{ix}&{n}_{iy}&{n}_{iz}&{\left({\boldsymbol{p}}_{i}\times{\boldsymbol{n}}_{\boldsymbol{i}}\right)}_{x}&{\left({\boldsymbol{p}}_{\boldsymbol{i}}\times{\boldsymbol{n}}_{\boldsymbol{i}}\right)}_{y}&{\left({\boldsymbol{p}}_{\boldsymbol{i}}\times{\boldsymbol{n}}_{\boldsymbol{i}}\right)}_{z}\\{s}_{ix}&{s}_{iy}&{s}_{iz}&{\left({\boldsymbol{p}}_{\boldsymbol{i}}\times{\boldsymbol{s}}_{\boldsymbol{i}}\right)}_{x}&{\left({\boldsymbol{p}}_{\boldsymbol{i}}\times{\boldsymbol{s}}_{\boldsymbol{i}}\right)}_{y}&{\left({\boldsymbol{p}}_{\boldsymbol{i}}\times{\boldsymbol{s}}_{\boldsymbol{i}}\right)}_{z}\\{a}_{ix}&{a}_{iy}&{a}_{iz}&{\left({\boldsymbol{p}}_{\boldsymbol{i}}\times{\boldsymbol{l}}_{\boldsymbol{i}}\right)}_{x}&{\left({\boldsymbol{p}}_{\boldsymbol{i}}\times{\boldsymbol{l}}_{\boldsymbol{i}}\right)}_{y}&{\left({\boldsymbol{p}}_{\boldsymbol{i}}\times{\boldsymbol{l}}_{\boldsymbol{i}}\right)}_{z}\end{array}\right].$$

The matrix ${J}_{i}$ presented here corresponds to the positional error. It is important to note that this framework is generic. If orientation accuracy is required for other applications, ${J}_{i}$ can be readily expanded to include rotational components (forming a $6\times6$ transformation matrix), and the subsequent matrix-based reliability evaluation remains applicable.

The pose error ${\boldsymbol{e}}_{i}$ is linearly related to the kinematic parameter error vector ${\boldsymbol{q}}_{i}$ through [14],

6 $$\boldsymbol{e}_{i}=\left[\begin{array}{c}d{x}_{i}\\d{y}_{i}\\d{z}_{i}\\\delta{x}_{i}\\\delta{y}_{i}\\\delta{z}_{i}\end{array}\right]=\left[\begin{array}{cccc}0&0&1&0\\{l}_{i}\cos{\alpha}_{i}&0&\sin{\alpha}_{i}&0\\-{l}_{i}\sin{\alpha}_{i}&0&\cos{\alpha}_{i}&0\\0&0&0&1\\\sin{\alpha}_{i}&0&0&0\\\cos{\alpha}_{i}&0&0&0\end{array}\right]\left[\begin{array}{c}\Delta{\theta}_{i}\\\Delta{l}_{i}\\\Delta{d}_{i}\\\Delta{\alpha}_{i}\end{array}\right]=\boldsymbol{I}_{i}\boldsymbol{q}_{i}.$$

By analyzing the matrix ${\boldsymbol{I}}_{i}$, it can be observed that the coefficients in the rows corresponding to orientation errors (the last three elements of ${\mathbf{e}}_{\mathrm{i}}$) are zero for the columns associated with linear parameters ${\Delta}{l}_{i}$ and ${\Delta}{d}_{i}$. This mathematically confirms that manufacturing errors in link lengths and offsets affect only the position of the end-effector and do not induce orientation errors. It should be noted, however, that this conclusion is valid only for serial mechanisms. For parallel mechanisms, dimensional errors in link lengths and offsets may affect the end-effector orientation due to kinematic coupling introduced by closed-loop constraints. The herein study focuses on simultaneous dimension and tolerance design of serial robotic mechanisms accounting for manufacturing cost and positioning accuracy.

### Positioning accuracy reliability evaluation

Considering the kinematic parameter errors from all links, it is convenient to assemble these errors into a unified vector form for further calculation. The overall error can be expressed as $\boldsymbol{Q}={[{\boldsymbol{q}}_{1},{\boldsymbol{q}}_{2},\dots,{\boldsymbol{q}}_{K}]}^{\mathrm{T}}$. Accordingly, the cumulative end-effector positional error can be written in compact matrix form,

7 $$\varDelta\boldsymbol{P}=\boldsymbol{H}\boldsymbol{Q},$$

where $\boldsymbol{H}={\left[{\boldsymbol{h}}_{1},{\boldsymbol{h}}_{2},\dots,{\boldsymbol{h}}_{K}\right]}_{3\times{K}}$ is the positional error coefficient matrix determined by the kinematic structure of robot. To quantitatively evaluate the positioning accuracy, the performance function is defined as the Euclidean norm of the end-effector positional error,

8 $$E\left(\boldsymbol{Q}\right)=\sqrt{{\Delta}{\boldsymbol{P}}^{\mathrm{T}}{\Delta}\boldsymbol{P}}=\sqrt{{\boldsymbol{Q}}^{\mathrm{T}}{\boldsymbol{H}}^{\mathrm{T}}\boldsymbol{H}\boldsymbol{Q}},$$

where $E\left(\boldsymbol{Q}\right)$ is the performance function of positioning accuracy due to kinematic parameter errors $\boldsymbol{Q}$.

Based on the established positioning error model, the reliability of positioning accuracy is defined as the probability that the end-effector positional error remains within the allowable limit. Specifically, failure probability of the position accuracy can be expressed as,

9 $${p}_{f}=P\left\{E\left(\boldsymbol{Q}\right)>r\right\},$$

where r is the allowable error, ${p}_{f}$ is the failure probability.

In summary, the positioning accuracy reliability model provides a quantitative means to evaluate how the joint parameter errors affect the positional deviation of the end-effector. By ensuring that the failure probability across all sampled target points remains below a specified threshold, the positioning reliability of the robot can be systematically controlled. This reliability criterion will be integrated into the optimization method as one of the objective functions, guiding the allocation of joint dimensions and tolerances to achieve both high positioning accuracy and low manufacturing cost.

## Dimension-tolerance-cost modeling

This section focuses on modeling the interrelated effects of joint dimensions, manufacturing tolerances, and manufacturing cost. Tighter tolerances typically increase manufacturing difficulty, especially when constrained by component size. To support cost-aware tolerance design, the section first describes how tolerance-induced errors are modeled, followed by a dimension-dependent cost formulation that reflects manufacturing feasibility.

### Modeling of tolerance-related errors

Specifically, the achievable tolerance level of each link is constrained by its dimensional parameters, and tighter tolerance generally requires higher manufacturing precision, leading to increased production costs. By formulating a cost model that incorporates these dependencies, the integrated optimization method can balance positioning accuracy and manufacturing feasibility.

In this study, the kinematic parameter errors are primarily attributed to manufacturing imperfections, such as dimensional deviations introduced during machining and assembly processes. Each kinematic parameter error ${\boldsymbol{q}}_{i}$ is assumed to arise from manufacturing variability. These errors are considered to follow independent Gaussian distributions due to the stochastic nature of the manufacturing process. Specifically, the error component $w$ in ${\boldsymbol{q}}_{i}$ is modeled as:

10 $$w\sim\mathcal{N}(0,{\sigma}_{w}^{2}),$$

where ${\sigma}_{w}$ is the standard deviation of the dimensional error. According to the widely adopted three-sigma principle in manufacturing quality control, the assigned manufacturing tolerance ${\tau}_{w}$ is directly related to the standard deviation:

11 $${\sigma}_{w}=\frac{{\tau}_{w}}{3}.$$

### Dimension-dependent cost model

In practical manufacturing, the achievable tolerance of a component is inherently influenced by its nominal dimension. International standards, such as ISO 286, provide systematic guidelines for assigning tolerances based on the nominal size of the part. However, most existing tolerance allocation methods focus solely on assigning dimensional tolerances in the design phase, without fully considering whether such tolerances are practically achievable during manufacturing, especially under the influence of the size-dependent tolerance limits. Ignoring this interdependence may result in infeasible or overly costly designs that require iterative redesign.

To address this gap, this study explicitly incorporates the size-dependent tolerance constraint into the overall cost function. By embedding the correlation between component dimensions, allowable tolerance levels, and manufacturing cost, the proposed model ensures that the allocated tolerances are not only optimal for positioning accuracy but also realizable within manufacturing capabilities associated with minimum cost.

Specifically, the manufacturing cost is modeled as a function of dimensions and assigned tolerances,

12 $$\mathrm{C}=\sum_{i=1}^{K}C\left({l}_{i},{\tau}_{i}\right),$$

where ${l}_{i}$ and ${\tau}_{i}$ denote the nominal length and tolerance of the $i-th$ link, respectively. The cost function $C({L}_{i},{\tau}_{i})$ is formulated to include:

13 $$C\left({L}_{i},{\tau}_{i}\right)=\frac{{C}_{1}{l}_{i}^{{C}_{2}}}{{\tau}_{i}^{{C}_{3}}}+{C}_{4}{l}_{i}.$$

where ${C}_{1}$, ${C}_{2}$ and ${C}_{3}$ are empirical coefficients, and ${C}_{4}$ material-dependent cost coefficient. The first term reflects the exponential increase in manufacturing difficulty and cost associated with tighter tolerances and larger dimensions [28]. The second term ${C}_{4}{l}_{i}$ represents the material cost that scales linearly with link length. This model provides a more flexible and continuous formulation compared to discrete ISO-grade-based cost coefficients, enabling a more accurate representation of size–tolerance–cost interactions in precision manufacturing. Note that this cost function only accounts for length-related tolerances but not angular tolerances.

The proposed cost function quantitatively captures the relationship between dimension, tolerance, and manufacturing cost, thereby enabling the integration of manufacturing feasibility into the structural design phase of robotic systems. Consequently, the optimization process naturally favors tolerance allocations that are within manufacturable limits while balancing positioning accuracy and production cost. This integrated approach effectively bridges the gap between idealized design parameters and actual manufacturing capabilities.

## Optimization method for tolerance allocation

### Matrix-based Monte Carlo simulations for reliability evaluation

To evaluate the positioning reliability across multiple workspace locations under geometric uncertainty, a matrix-based MCS method is proposed. Unlike conventional approaches that compute sample-based error propagation separately for each position, this method reformulates the reliability analysis into a batch matrix structure, enabling efficient parallel computation over all sampled configuration and Monte Carlo simulations.

Assume the manipulator consists of $K$ links, and each link contributes four independent kinematic error parameters. Let the total number of kinematic error sources be$4K$, denoted by the set $[{w}_{1},{w}_{2},\dots,{w}_{4f}]$. To quantify the influence of kinematic uncertainties on positioning accuracy, a subset of $S$ parameter error sources is selected from the total $4K$kinematic parameters. Each element in the kinematic parameter error follows a zero-mean Gaussian distribution ${w}_{s}\sim\mathcal{N}(0,{\sigma}_{{w}_{s}}^{2})$, $s\in\{\mathrm{1,2},\dots,S\}$, with variance derived from the tolerance model described in Sect.  3.2. To efficiently propagate the effect of these uncertainties to the end-effector, a global sampling matrix is constructed by drawing $N$ samples from the joint Gaussian distributions. For each error source, $N$ independent samples are drawn from its distribution, forming the individual sampling vector,

14 $${\boldsymbol{W}}_{\boldsymbol{s}}=\left[\begin{array}{c}{w}_{s}^{1}\\{w}_{s}^{2}\\⋮\\{w}_{s}^{N}\end{array}\right]\in{\mathbb{R}}^{N\times1},$$

where ${w}_{s}^{j}$ denotes the $j-th$sample of the $s-th$ error parameter. The sampled vectors from all $s$ error sources are then aggregated into a global sampling matrix $\mathcal{W}={\left[{\boldsymbol{W}}_{1}^{T},{\boldsymbol{W}}_{2}^{T},\dots,{\boldsymbol{W}}_{S}^{T}\right]}^{T}\in{\mathbb{R}}^{S\times{N}}$.

To ensure that positioning accuracy is satisfied throughout the entire workspace, the motion space is discretized with $M$ sampled configurations. Each configuration represents a specific point of the robot during operation. For each configuration $m\in\{1,2,\dots,M\}$, the corresponding positional error coefficient matrix is ${\boldsymbol{H}}_{m}\in{\mathbb{R}}^{3\times{S}}$. These matrices capture the sensitivity of the end-effector position to the selected kinematic parameter deviations at different configurations. By stacking them, a three-dimensional error propagation tensor is constructed,

15 $$\mathcal{H}={\left[\begin{array}{c}{\boldsymbol{H}}_{1},{\boldsymbol{H}}_{2},\dots,{\boldsymbol{H}}_{M}\end{array}\right]}^{T}\in{\mathbb{R}}^{M\times3\times{S}}.$$

This tensor enables efficient batch computation of propagated errors across all configurations using the same global errors sampling matrix $\mathcal{W}$ of kinematic parameter. Based on the previously defined error coefficient tensor $\mathcal{H}\in{\mathbb{R}}^{3\times{S}\times{M}}$ and the global errors sampling matrix $\mathcal{W}\in{\mathbb{R}}^{S\times{N}}$, the end-effector positional errors at all $M$ sampled configurations under $N$ error samples can be computed simultaneously. To streamline the computation across all positions, a matrix multiplication is applied between $\mathcal{H}$ and $\mathcal{W}$,

16 $$\mathcal{P}=\mathcal{H}\mathcal{W}\in{\mathbb{R}}^{M\times3\times{N}},$$

Here, each slice ${\boldsymbol{P}}_{m}\in{\mathbb{R}}^{3\times{N}}$ in $\mathcal{P}=[{\boldsymbol{P}}_{1},{\boldsymbol{P}}_{2},\dots,{\boldsymbol{P}}_{M}]$ represents the sampled 3D positional deviations at configuration $m$. The Euclidean norm of each column in ${\boldsymbol{P}}_{m}$ yields the error magnitudes for all samples, formulated as:

17 $${\mathbf{E}}_{i}^{k}={∥{\mathcal{P}}_{i,:,k}∥}_{2}=\sqrt{\sum_{j=1}^{3}{\left({\mathcal{P}}_{i,j,k}\right)}^{2}}.$$

Given an allowable threshold $r$, the positioning failure probability at each configuration is defined as the ratio of samples that exceed the allowable error limit,

18 $$\boldsymbol{F}=\frac{1}{N}\sum_{k=1}^{N}\mathbb{I}\left({∥{\mathcal{P}}_{i,:,k}∥}_{2}\ge{r}\right),\boldsymbol{F}\in{\mathbb{R}}^{M},$$

where $\mathbb{I}$ is the indicator function, returning 1 if the condition holds, and 0 otherwise. ***F*** is failure probability in all sampled configurations. Accordingly, the positioning reliability $\boldsymbol{R}\in{\mathbb{R}}^{M}$ of all configurations can be expressed as $\boldsymbol{R}=1-\boldsymbol{F}$. This formulation enables efficient batch evaluation of positioning reliability for workspace through matrix operations, providing a foundation for integrating reliability constraints into the optimization method.

### Multi-objective formulation and design parameters selection

To achieve a balance between high positioning accuracy and manufacturability, a multi-objective optimization method is established. The objectives are to minimize the maximum failure probability of positioning accuracy—quantified by workspace positioning reliability—and the manufacturing cost associated with joint dimensions and tolerances.

The multi-objective optimization problem can be expressed as:

19 $$\left\{\begin{array}{c}\begin{array}{c}\underset{x}{\mathrm{m}\mathrm{i}\mathrm{n}}\underset{M}{\mathrm{m}\mathrm{a}\mathrm{x}}\left(F\right)\\\\\underset{x}{min}\sum_{i=1}^{h}C({l}_{i},{\tau}_{i})\\s.t.{w}_{s}\sim{N}(0,{\tau}_{{w}_{s}}^{2}/9)\\\\{\tau}_{{w}_{s}}^{min}\le{\tau}_{{w}_{s}}\le{\tau}_{{w}_{s}}^{max}\end{array}\end{array}\right.$$

Then, optimization algorithm (e.g., genetic algorithms) could be used to optimize the multi objective problem. The final solution can be selected from the Pareto front based on design constraints, performance priorities, or manufacturing capabilities. In summary, the multi-objective tolerance and dimension design process is outlined as follows:

- **Step 1**. Define the design variables, including selected link lengths and its associated tolerance values. Initialize the population with random samples within the feasible bounds.
- **Step 2**. For each individual with specified of dimensions and tolerances in the population, construct the error sampling matrix $\mathcal{W}$ based on Gaussian distributions with standard deviations derived from the corresponding tolerances.
- **Step 3**. Compute the positional error coefficient tensor $\mathcal{H}$ for all sampled workspace configurations.
- **Step 4**. Calculate the propagated positional errors and evaluate the positioning reliability $R$ at each sampled configuration using matrix-based methods.
- **Step 5**. Evaluate the objective functions: (1) minimize the maximum failure probability (maximize reliability), and (2) minimize the total manufacturing cost as defined in Sect.  3.2.
- **Step 6**. Apply optimization operators to evolve the population: perform crossover, mutation, and selection.
- **Step 7**. Repeat Steps 2–6 until the maximum generation is reached or convergence criteria are satisfied. Output the Pareto-optimal solutions for dimension and tolerance allocation.

## Case study on dimension and tolerance optimization of a new surgical robot

**Fig. 2:**
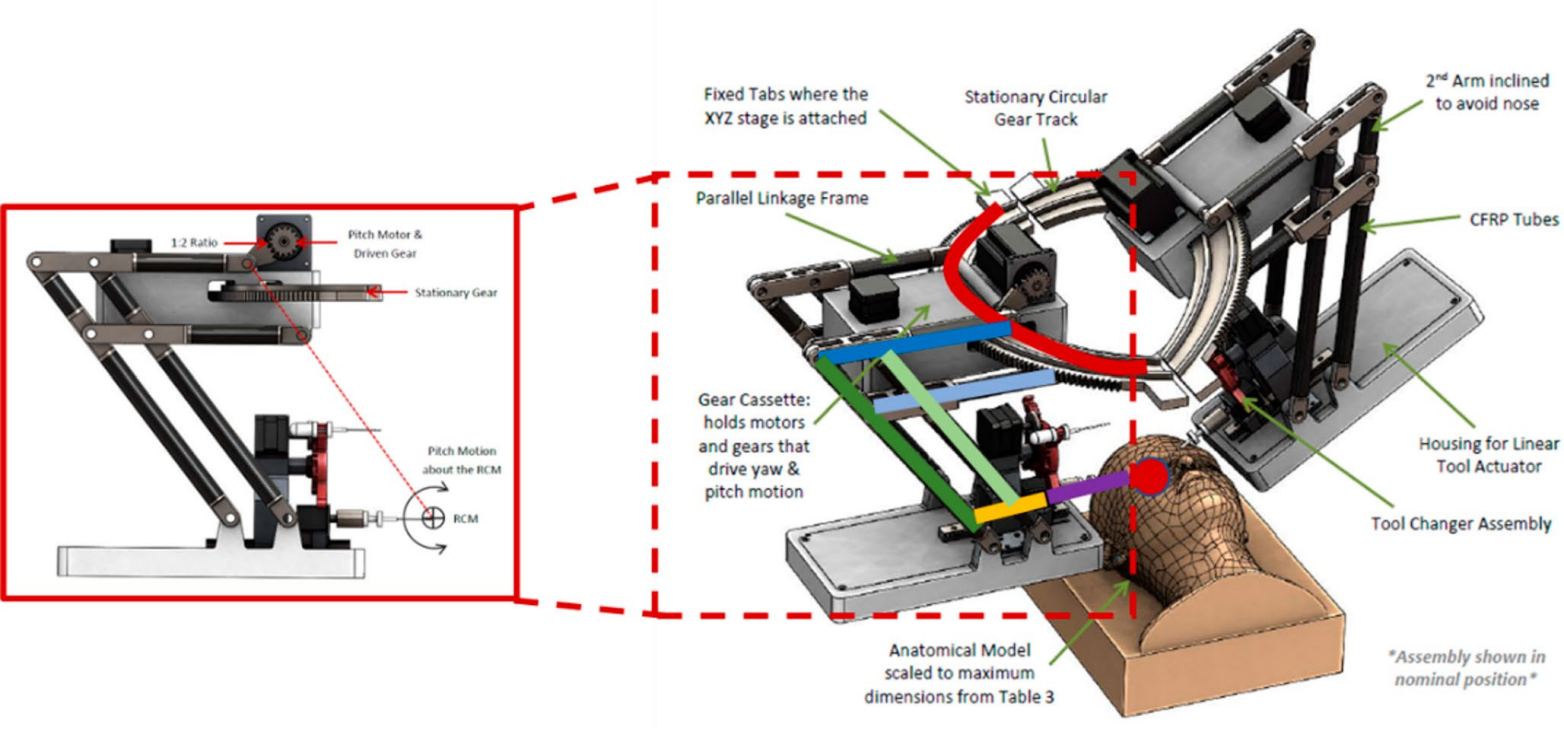
Physical structure of the RCM mechanism integrating a parallel linkage frame and circular gear track

This section presents a case study to validate the proposed integrated tolerance optimization method using a surgical robot with a mechanically constrained RCM mechanism, as shown in Fig. 2. The case focuses on analyzing the impact of link dimensional parameters and manufacturing tolerances on the positioning accuracy, under the constraints of surgical precision and manufacturability. First, the structure and motion characteristics of the robot are introduced. Then, a simplified kinematic model is established to support error propagation and optimization. Finally, multi-objective optimization is conducted, and the results are analyzed to demonstrate the effectiveness of the proposed method in balancing accuracy and manufacturing cost.

### Mechanism description

The manipulator is configured to provide 4 degrees of freedom (DoFs) at the tool tip relative to the RCM. It is constructed by a stationary circular gear track on the base, followed by parallelogram mechanism to provide the pitch motion at a mounting platform. This arrangement enables 65° pitch and ± 45° yaw rotations, allowing sufficient motion range during surgical operation while maintaining the RCM constraint. A linear actuator is mounted to control the translational insertion motion of the surgical tool along the tool axis, providing up to 10 mm range and continuous roll motion about the tool axis is achieved at the distal end.

### Kinematic modeling with link parameters

**Fig. 3:**
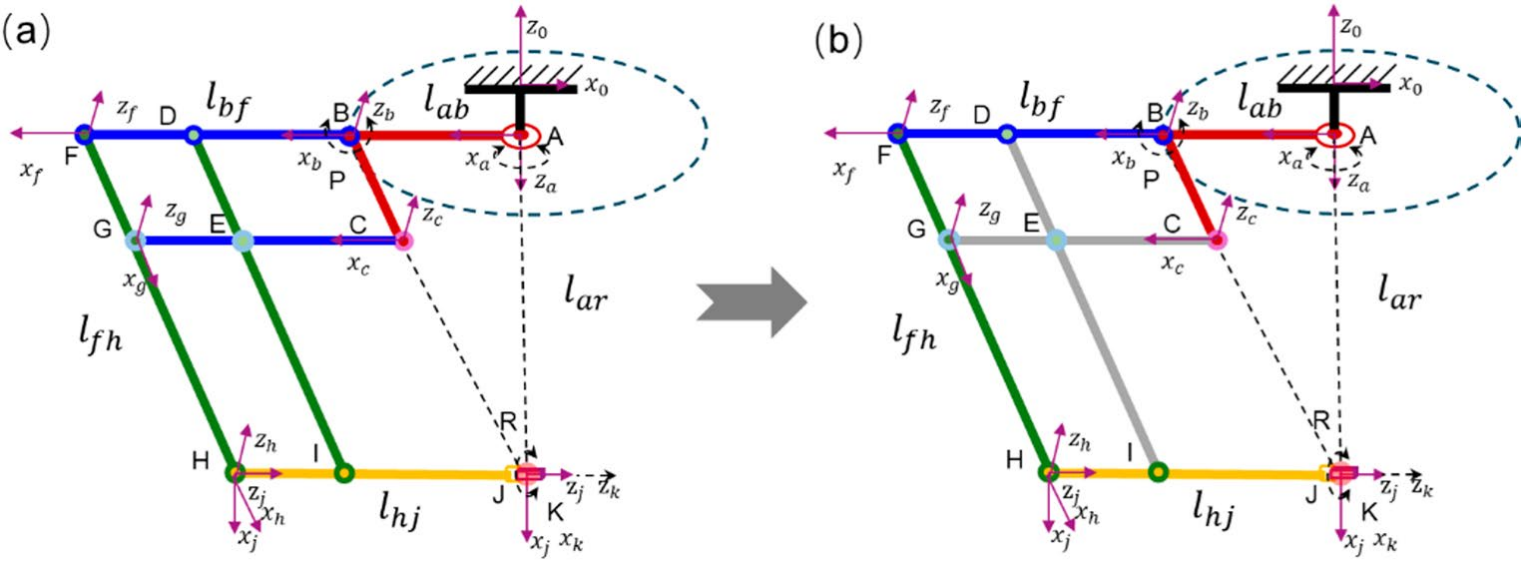
Kinematic description of the RCM mechanism

Figure 3 (a) shows the kinematic description of the robot mechanism. The circular gear track and yaw motion are represented by a fixed revolute joint (A) at the base. The planar parallel linkage frame is simplified into four-bar linkages (B to I) enabling pitch motion about the RCM point. The distal section, responsible for tool insertion and roll, is modeled by a prismatic joint (J) and a revolute joint (K).

It is worth noting that the RCM mechanism contains a parallelogram-based parallel structure, whose primary function is to impose geometric constraints, i.e., the two pairs of opposite sides always parallel to each other (i.e., BF // HK, FH // BK) to maintain the position of the remote center of motion. Meeting these parallel constraints is always the first priority in fabrication and assembly as failing to achieve it will result in assembly difficulties, orientation errors, and even invalid kinematic mode [29]. The error budget of parallel mechanisms is not the focus of this herein study, and the parallelogram structure is simplified into an equivalent serial kinematic chain associated with these parallel constraints. Therefore, the position errors of joints B, F, and H (as shown in Fig. 3) are accounted for this study. Because two of the links primarily serve to maintain the RCM property and do not introduce independent motion, these are omitted in the simplified kinematic chain (Fig. 3 (b)), and their effect is embedded as geometric constraints. The parallelogram structure inherently possesses one degree of freedom, with joint B as the only actuated input. The motions of other joints (e.g., H and F) are passively determined by the linkage constraints. To enable a simplified serial representation of the mechanism while maintaining its essential motion characteristics, two geometric constraints are transformed into constraints in D-H parameters of the mechanism. The first constraint is the introduction of an initial reference angle ${\theta}_{0}$, defined as the angle ∠ABC when the distal link HR is aligned horizontally. This parameter represents the reference configuration of the parallelogram and allows the dependent joint angles to be expressed as functions of ${\theta}_{b}$and ${\theta}_{0}$. The second constraint is a geometric length constraint defined in the reference configuration, where the distal link HR is horizontal. In this configuration, the links ${l}_{ar}$, ${l}_{ab}$ and ${l}_{fh}$ form a triangle, with the angle between ${l}_{ab}$ and ${l}_{fh}$​ equal to the initial reference angle ${\theta}_{0}$.

Based on the above joint configuration and constraint relationships, the forward kinematic model of the RCM mechanism is established using forward D-H method, and the corresponding D-H parameters for the mechanism is summarized in Table 1.

**Table 1:** D-H parameters of mechanism

Joint	Number	Joint angle ($\theta$)	Joint offset ($d$)	Link twist ($\alpha$)	Link length ($l$)	Range
A	1	0	0	pi	$0$	-
B	2	${\theta}_{a}$	0	$3/2*\pi$	${l}_{ab}$	$\pm45^\circ$
F	3	${\theta}_{b}$	0	0	${l}_{bf}\text{}$	$65^\circ$
H	4	$\pi+{\theta}_{0}-{\theta}_{b}$	0	0	${l}_{fh}$	-
J	5	$\pi/2-{\theta}_{0}+{\theta}_{b}$	0	$\pi$/2	0	-
K	6	${\theta}_{k}$	${l}_{hj}+D$	0	0	$360^\circ/10mm$

### Optimization results and analysis

The optimization is performed based on the idealized kinematic model. The link parameters include the kinematic parameters of three structural links $\left({l}_{ab},{l}_{bf},{l}_{fh},{l}_{hj}\right)$ and the corresponding tolerance $\boldsymbol{\tau}=({\tau}_{ab},{\tau}_{bf},{\tau}_{fh},{\tau}_{hj})$. Due to the mechanical constraints that the lengths $\left({l}_{ab}{,l}_{fh}\right)$ and $({l}_{bf},{l}_{hj})$ must satisfy constrains mentioned above, only two link lengths and four tolerance-related parameters are selected as independent variables in the optimization process. Therefore, four link parameter errors $\boldsymbol{W}=(\varDelta{l}_{2},\varDelta{l}_{3},\varDelta{l}_{4},\varDelta{l}_{6})$ are modeled as the random variables ${w}_{s}\sim{N}\left(0,{\sigma}_{s}^{2}\right),s\in\{\mathrm{1,2},\mathrm{3,4}\}$, in which ${\sigma}_{{w}_{s}}={\tau}_{{w}_{s}}/3$.

**Fig. 4:**
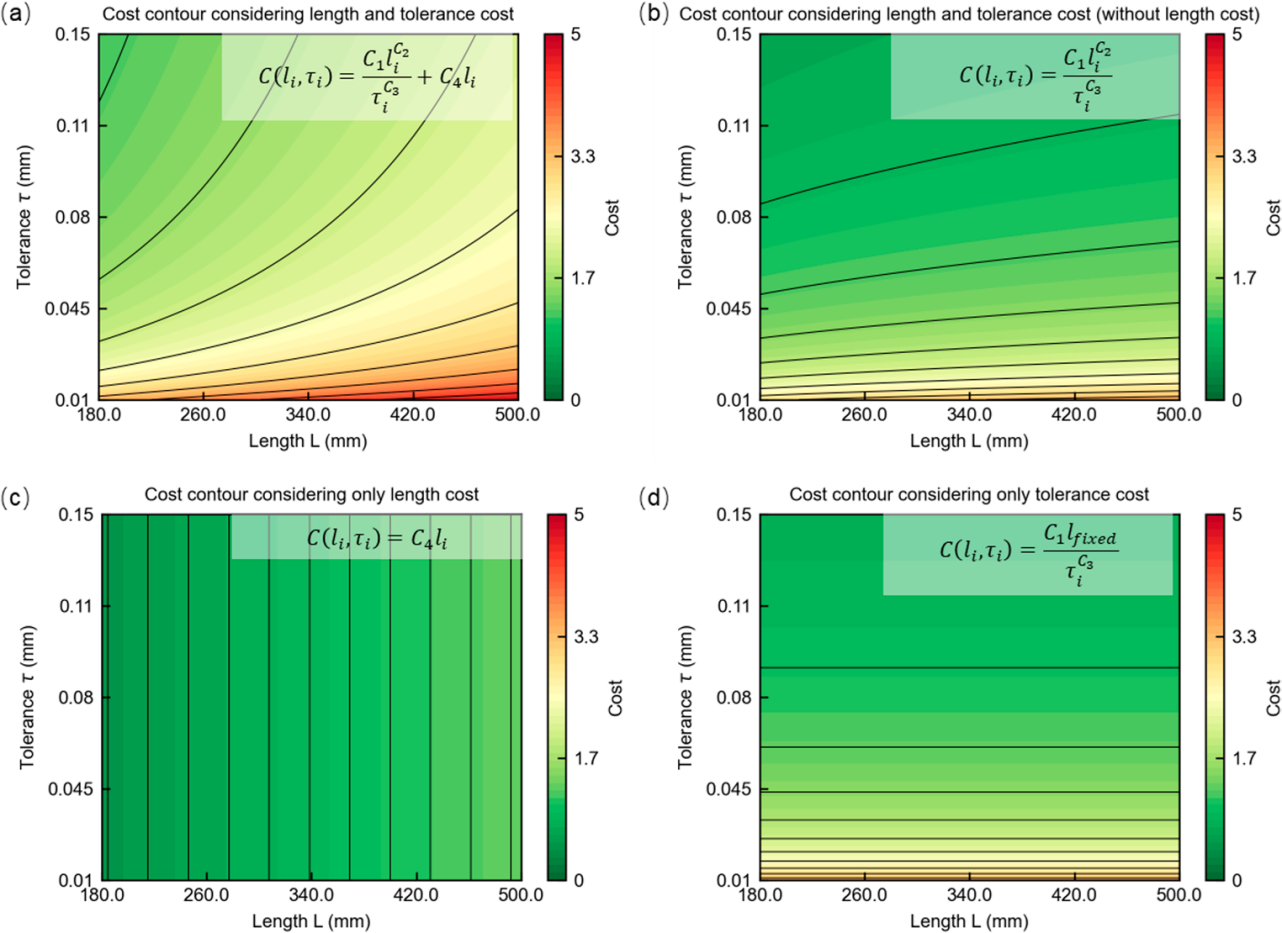
Cost contour with respect to length and tolerance (**a**) Cost contour with respect to length and tolerance. (**b**) Cost contour with respect to length and tolerance without the cost item of length cost. (**c**) cost contour with respect to length cost. (**d**) const contour with respect to tolerance cost at a fixed dimension ${l}_{fixed}$

Considering that the application target of this study is robotics, ISO 286 is adopted as the reference standard for tolerance classification and cost modeling, which is developed for high-end manufacturing fields such as robotics and aerospace. The standard defines 13 nominal size ranges and 20 tolerance grades, commonly referred to as IT (International Tolerance) grades. Based on engineering practices, the tolerance grades for all link parameters are preliminarily selected within the ISO IT2–IT11 range. Specifically, the cost function is derived from the exponential relationship suggested in reference [28], which relates the tolerance level to manufacturing complexity, and the following coefficients are adopted: ${C}_{1}=0.0896$, ${C}_{2}=0.183$, and ${C}_{3}=0.55$. Considering that tolerance-related and material costs are approximately equal for an IT7-level tolerance at a nominal length of 500 mm, and assuming that material cost scales linearly with component length, the length-dependent coefficient is set as ${C}_{4}=0.0971$. The relationship among dimension, tolerance, and cost is illustrated in the Fig. 4.

In Fig. 4(a), the length-tolerance related cost are considered, which shows that the cost increases sharply when tighter tolerances are required, and increases more gradually when the link length grows. This illustrates a realistic coupled relationship in which both geometric size and precision requirements jointly influence manufacturing cost. producing a coupled cost landscape. In Fig. 4(b), the linear length term is removed, leaving only the length -tolerance-driven component $\frac{{C}_{1}{l}_{i}^{2}}{{\tau}_{i}^{{C}_{3}}}$. As a result, for high-tolerance (small ${\tau}_{i}$) regions the cost surface becomes relatively flat, indicating that loose tolerances are dominated by the tolerance-dependent term and thus less sensitive to length variation. However, as tolerances become loose, the influence of link length gradually emerges, reflected in the increasing slope of iso-cost contours along the ${L}_{i}$direction. Figure 4(c) shows the case where only the length-related term ${C}_{4}{l}_{i}$ is retained. Figure 4(d) presents the cost model frequently used in existing literature, where only the tolerance term is considered and ignores the length influence on tolerance. Although this formulation captures the basic effect of tolerance tightening, it neglects the fact that manufacturing the same tolerance on longer components is inherently more difficult and costly. Consequently, the cost contours appear entirely horizontal, implying identical cost across different lengths. Comparing Fig. 4(a) with Fig. 4(d) reveals an important limitation of the traditional tolerance-only model that it fails to account for the coupling between tolerance and link size, leading to noticeable bias in cost estimation when link dimensions vary. By contrast, the full model in Fig. 4(a) provides a more realistic mapping of manufacturing cost, which is essential for accurate multi-objective optimization involving both dimensions and tolerances.

Furthermore, considering the parallelogram mechanism structure of the manipulator, the cost estimation additionally includes the influence of link lengths ${l}_{cg}$ and ${l}_{di}$. To ensure geometric consistency, ${l}_{cg}$ shares the same length and tolerance parameters as ${l}_{bf}$, while ${l}_{di}$ set to match ${l}_{fh}$.

**Fig. 5:**
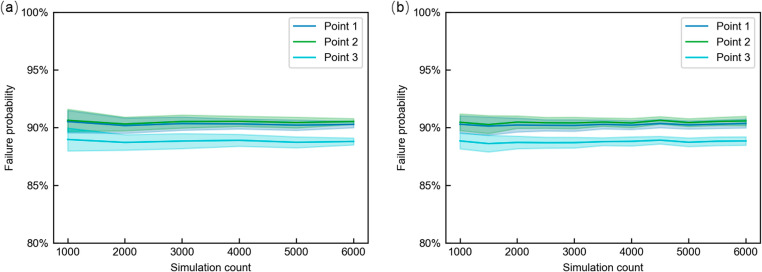
Statistical Convergence of Failure Probability for 3 Sample Configurations. (**a**) Convergence of failure-probability estimation using Monte Carlo sampling. (**b**) Convergence of failure-probability estimation using Latin Hypercube Sampling (LHS)

The workspace analysis primarily considers the variations introduced by variables ${\theta}_{a}$ (yaw), ${\theta}_{b}$(pitch), and $D$ (insertion depth), while ${\theta}_{k}$ accounts for the tool’s rotational motion and is excluded from spatial sampling. To evaluate the positioning accuracy reliability, the joint angles are sampled at 3° intervals and the prismatic joint D is sampled at 1 mm intervals, resulting in a total of 6600 configurations. To determine a sufficient number of MCS for reliable estimation, three representative configurations were randomly selected. For configuration, the convergence behavior was evaluated by varying the number of samples from 1,000 to 6,000. At each sampling level, 30 independent trials were conducted to observe the distribution of the estimated failure probabilities. In addition to MCS, the convergence of Latin Hypercube Sampling (LHS) was evaluated using the same procedure to provide a variance-reduced baseline for comparison. The results are illustrated in Fig. 5.

Figure 5 compares the convergence behavior of the failure-probability estimation when using MCS (Fig. 5 (a)) and LHS (Fig. 5 (b)). As shown in Fig. 5(a), when the number of MCS exceeds 4,000, the variation in failure probability remains within 1%, and the results exhibit stable convergence. Therefore, 4,000 MCSs are performed at each workspace sampling point following error distributions to estimate positioning accuracy reliability. In contrast, Fig. 5 (b) shows that LHS achieves stable estimation with fewer samples, approximately 3000 samples are sufficient for all three points to fall within the same 1% deviation band. This improvement results from stratified sampling in LHS, which enhances the coverage of the input space and reduces estimator variance.

**Fig. 6:**
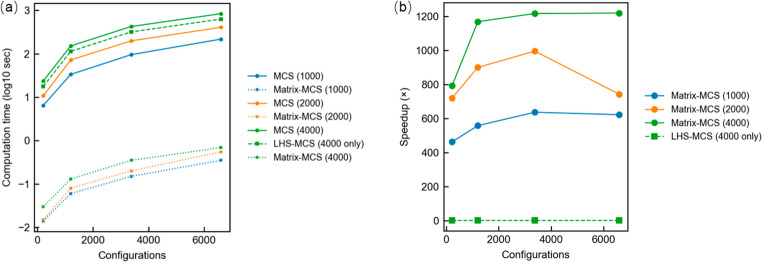
Comparison of computation time between conventional MCS and matrix-based MCS for full workspace reliability evaluation

To verify the computational efficiency of the proposed matrix-based MCS method, a comparison was conducted against the conventional MCS approach and LHS approach. As shown in Fig. 6(a), when the number of configuration or samples is small, the conventional MCS method remains relatively manageable; however, as the problem size scales up, its computation time grows rapidly due to the need to perform repeated individual kinematic evaluations. In contrast, the matrix-based method maintains its efficiency by leveraging vectorized operations to simultaneously compute positional errors across all samples and configurations. Figure 6(a) also shows that LHS-MCS, while providing lower variance in probability estimation, does not reduce computational time.

The computational advantage of the proposed matrix-based MCS method becomes increasingly pronounced with the growth of both sampling configurations and MCS sample size. As illustrated in Fig. 6(b), the speedup reaches more than 600× for 1000 samples, exceeds 800× for 2000 samples, and surpasses 1200× for 4000 samples when evaluating the full 6600-configuration workspace. This trend demonstrates excellent scalability with respect to both the number of samples and the number of configurations. In contrast, LHS-MCS shows negligible speedup and even slight slowdown due to its additional sampling cost. These results highlight that the proposed matrix-based MCS approach is highly efficient and well suited for high-resolution reliability evaluation in robotic design optimization.

Based on practical application requirements, the allowable positional accuracy threshold for the robotic system is set to 0.050 mm. The joint parameter tolerances are constrained within the range of 0.010–0.150 mm, and the link lengths are bounded between 200 mm and 500 mm. Additionally, the positioning failure probability must not exceed 0.10. Under these constraints, the complete tolerance design model is formulated as follows:

20 $$\left\{\begin{array}{c}\underset{x}{min}\underset{M}{max}\left({R}^{6600}\right)\\min\sum_{i=1}^{6}C\left({L}_{i},{\tau}_{i}\right)\\{w}_{s}\sim{N}\left(0,\frac{{\tau}_{s}^{2}}{9}\right),s\in\{\mathrm{1,2},\dots,4\}\\\\\begin{array}{cc}\mathrm{s}.\mathrm{t}.&0.010mm\le{\tau}_{{w}_{s}}\le0.150\mathrm{m}\mathrm{m}\\\\&200mm\le{L}_{i}\le500\mathrm{m}\mathrm{m}\\\\&\underset{M}{max}\left({R}^{6750}\right)\le0.10\end{array}\end{array}\right.$$

**Fig. 7:**
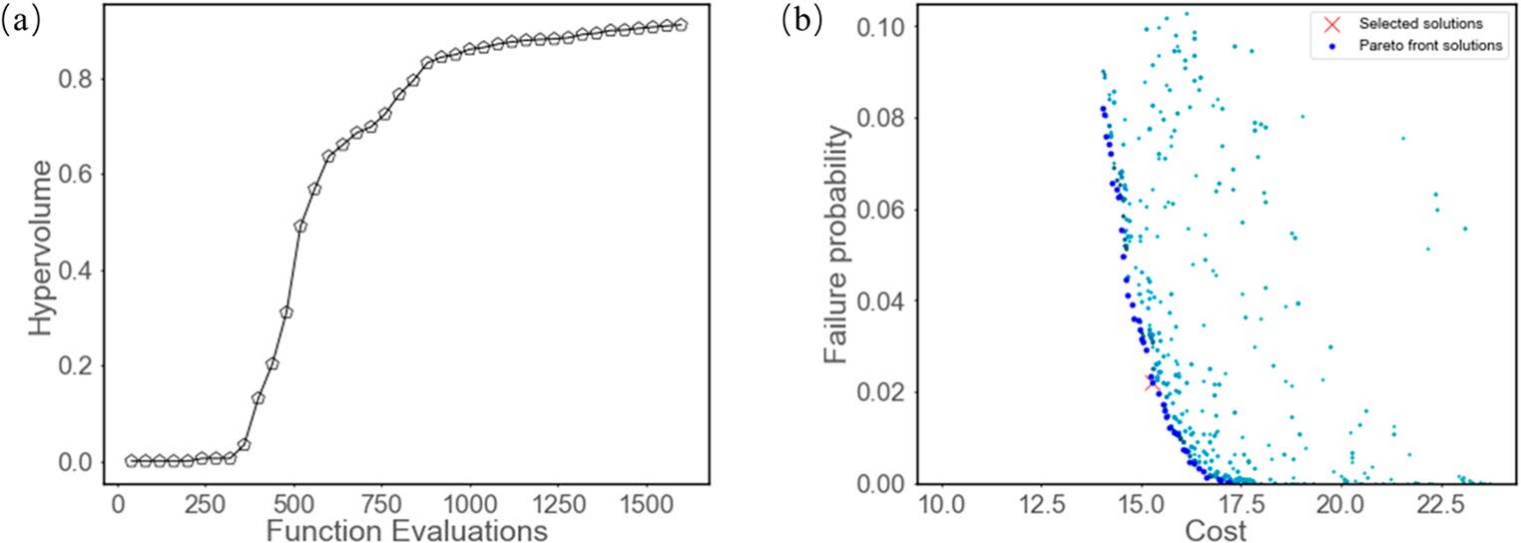
Optimization results. (**a**) Hypervolume convergence. (**b**) Pareto front of optimization results

The NSGA-II multi-objective optimization algorithm is employed to solve the above tolerance design model. The population size is set to 20, and the number of generations is set to 60. The optimization results are illustrated in Fig. 7.

From Fig. 7 (a), the optimization process shows clear convergence. A set of Pareto-optimal solutions is obtained, as illustrated in Fig. 7 (b), where feasible configurations meeting the reliability constraint exhibit cost values ranging from 14 to 18. Using the entropy evaluation method [30], an optimal solution is selected from the Pareto set, as indicated in Fig. 7 (b). A comparison is also conducted with an existing model [23] that considers only the tolerance-cost relationship(T-C model) with pre-determined length parameters, in which all joint errors are assumed to follow the IT7 tolerance grade. Both approaches use the same optimization algorithm, parameter settings, and entropy-based solution selection method. The only difference lies in the cost model with or without link dimensions. The configurations of the optimal solutions obtained from the two methods are summarized in Table 2.

**Table 2:** Optimized parameters of selected solution

Method	${l}_{ab}$ (mm)	${l}_{bf}$ (mm)	${\tau}_{ab}$ (um)	${\tau}_{bf}$ (um)	${\tau}_{fh}$ (um)	${\tau}_{hj}$ (um)	Failure probability	Cost
The proposed method	203	201	15	33	29	63	0.022	**15.28**
Tolerance grade	-	-	**IT4**	**IT6**	**IT5**	**IT7**	-	
T-C model	400	600	18	35	26	58	0.027	19.50
Tolerance grade	-	-	**IT4**	IT5	IT4	IT6	-	

As observed in Table 2, the proposed method achieves a comparable failure probability (0.022) to the traditional method using a T-C model (0.027) while reducing the overall cost from 19.50 to 15.28, which is a reduction of approximately 22%. This demonstrates its ability to maintain reliability with significantly improved cost efficiency. Notably, by explicitly incorporating link dimension constraints into the optimization, the proposed method tends to allocate larger tolerance values overall, leading to higher tolerance grades (e.g., IT4 to IT7), which are generally more economical to manufacture. For the four commonly optimized tolerance parameters (${\tau}_{ab}$, ${\tau}_{bf}$, ${\tau}_{fh}$ and ${\tau}_{hj}$), the proposed method yields mixed outcomes in terms of numerical tolerance values. Although the tolerance values of ${\tau}_{ab}$ and ${\tau}_{bf}$ are slightly lower than those in the T-C model, their corresponding IT grades are higher (i.e., IT4 and IT6 vs. IT4 and IT5), indicating a more realistic and practical choice that aligns with feasible manufacturing standards. In contrast, the T-C model, while initially assuming IT7 for all tolerances, produces an optimized solution with some tolerances falling into tighter and potentially costlier ranges. This outcome suggests that without considering the dimension–tolerance dependency, the optimization tends to over-constrain tolerances to minimize positioning error, potentially leading to higher production cost and impractical manufacturing requirements. By integrating dimension-aware tolerance feasibility, the proposed method provides a more realistic and cost-effective solution.

**Fig. 8:**
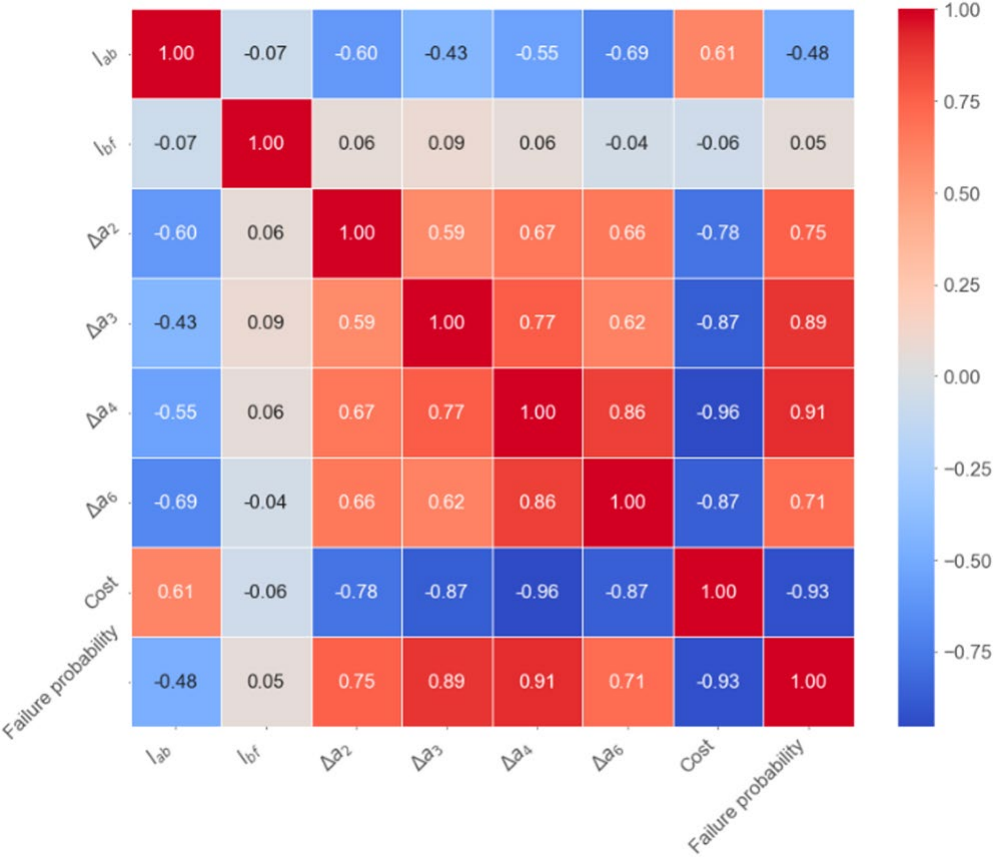
Correlation of design parameters, cost, and failure probability derived from Pareto-optimal solutions

A correlation analysis based on Pearson correlation is conducted on the Pareto-optimal solutions obtained by the proposed method to reveal the interdependence between dimensional parameters, cost, and positioning reliability. As shown in Fig. 8, in addition to the expected trends, such as the negative correlation between failure probability and cost, the negative correlation between tolerance values and cost, and the positive correlation between tolerance values and failure probability, some notable insights emerge from the analysis. Specifically, the tolerance parameters $\varDelta{l}_{3}$ and $\varDelta{l}_{4}$ exhibit significantly stronger positive correlations with failure probability (0.89 and 0.91) compared to $\varDelta{l}_{2}$ and $\varDelta{l}_{6}$ (0.75 and 0.71). This indicates that the positioning accuracy of the two joints in the parallel linkage structure plays a more critical role in determining the overall positioning reliability of the mechanism. Therefore, a parallelogram linkage is employed to mitigate the propagation of dimensional errors from these critical joints. The symmetrical geometry and kinematic redundancy inherent in parallel structures help localize error effects and enhance the overall positioning reliability of the mechanism.

**Fig. 9:**
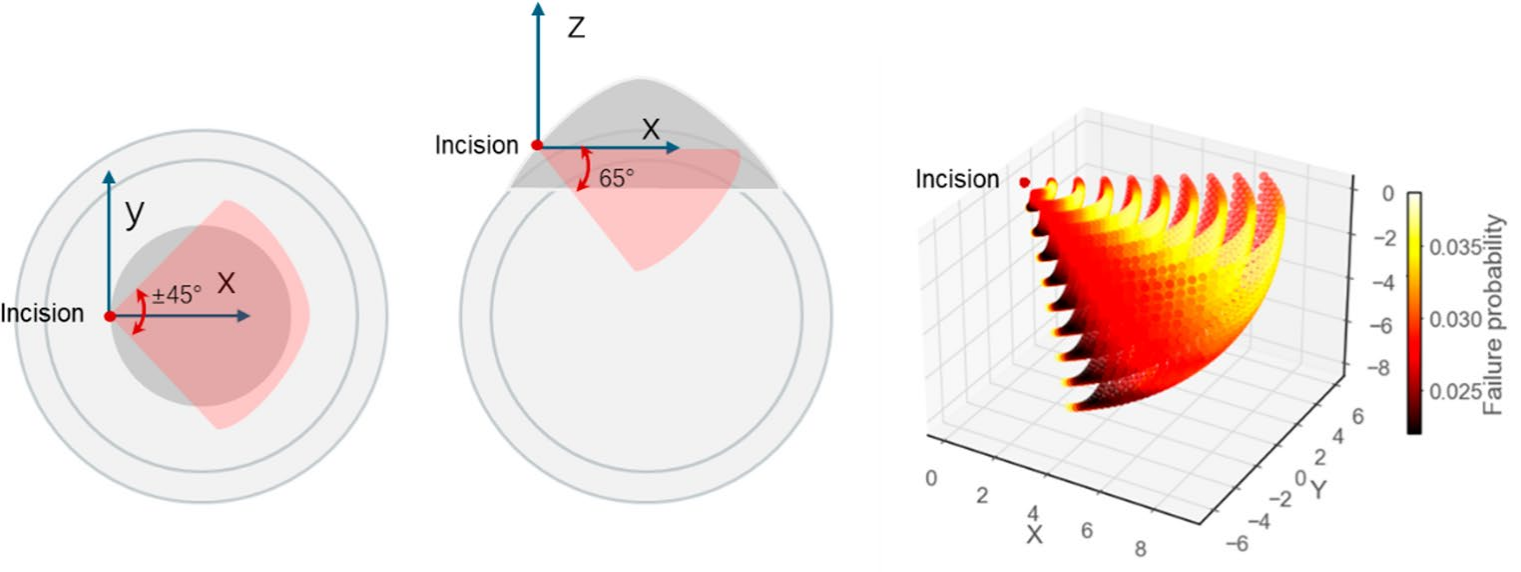
Failure probability distribution across the robot workspace

The failure probability distribution within the workspace is illustrated in Fig. 9. It can be observed that most points exhibit failure probabilities in the range of 0.025 to 0.036, where the maximum failure probabilities is 0.036. Regions with relatively higher failure probabilities are concentrated in the inner areas of the workspace, while the outer workspace regions demonstrate lower failure risks. This distribution pattern indicates that the optimized tolerance configuration effectively satisfies the reliability requirements for surgical robotic applications, where workspace boundaries are typically critical for task execution.

## Discussion and conclusion

This paper proposes a novel simultaneous dimension and tolerance design approach that explicitly incorporates the relationship between dimensional size and achievable tolerance, enabling simultaneous optimization of manufacturing cost and positioning accuracy reliability. By constructing a cost model that couples link dimensions tolerance, and cost, the proposed method accounts for both tolerance difficulty and part dimensions, leading to reduced manufacturing cost and manufacturing complexity. To efficiently evaluate reliability across the full workspace, a matrix-based MCS method is developed. This approach enables batch computation of end-effector accuracy under uncertainty using matrix operations, achieving over 400 times speedup compared to conventional MCS while maintaining accuracy. A case study is conducted to verify the proposed method. The case study results demonstrate that the proposed simultaneous design method achieves comparable positioning reliability to traditional tolerance-cost models, while significantly reducing manufacturing cost by approximately 22% in the test case. Furthermore, by considering the dependency between link dimensions and tolerance grades, the method enables the use of looser tolerance classes (e.g., IT4 to IT7) that are more consistent with practical manufacturing capabilities, thereby reducing machining difficulty and improving cost-efficiency. These findings highlight the practical advantage of integrating dimension-tolerance coupling into the design process for high-precision robotic systems. Furthermore, in scenarios where orientation error is critical, the proposed method can be applied to evaluate orientation reliability and positional reliability separately using matrix-based MCS method. These metrics can then be optimized simultaneously as distinct objectives within the multi-objective optimization framework.

The current cost model focuses on dimensions and tolerance constraints for only serial robots. Future work will extend the error propagation model to account for all types of errors including joint assembly conditions for both serial and parallel robotic mechanisms.

## Data Availability

Data will be made available on request.
